# Assessment of community pharmacists’ knowledge, attitudes and their willingness to provide vaccination services in Saudi Arabia

**DOI:** 10.1371/journal.pone.0304287

**Published:** 2024-05-28

**Authors:** Abdulkarim M. Meraya, Mamoon H. Syed, Abdulwahab A. Shabi, Huthaifa A. Madkhali, Younis A. Yatimi, Khalied Y. Khobrani, Yahia A. Mubarki, Amani Khardali, Hilal Thaibah, Ayesha Yasmeen

**Affiliations:** 1 Department of Clinical Pharmacy, College of Pharmacy, Jazan University, Jazan, Saudi Arabia; 2 Pharmacy Practice Research Unit, College of Pharmacy, Jazan University, Jazan, Saudi Arabia; University of Hail, SAUDI ARABIA

## Abstract

**Background:**

Community pharmacists play an important role in increasing vaccination rates especially in countries where they offer vaccination services and administration. However, little is known about community pharmacist’s willingness to provide vaccination services in Saudi Arabia. The objective of this study was to assess knowledge, attitudes, willingness and beliefs of community pharmacists in Saudi Arabia towards providing vaccines at pharmacies.

**Methods:**

A cross-sectional, online questionnaire-based study using convenience sampling (Snowball technique) was used to obtain responses from community pharmacists across Saudi Arabia. The survey collected information on participants’ demographics, knowledge about vaccine, attitude towards vaccine and their attitude to be immunization providers. Bivariate analysis and multiple linear regression models were employed to assess the relationships between demographic variables and outcomes.

**Results:**

The study sample consisted of 384 community pharmacists. More than half of participants had poor knowledge about vaccines (54%). Only 8.4% of participants had good knowledge about vaccines. The results indicated that community pharmacists in the study sample have positive attitude toward vaccines and are willing to provide vaccination services. There was a significant relationship between knowledge about vaccine and attitude toward vaccines. Pharmacists with poor knowledge about vaccines had negative attitude toward vaccines as compared to those with high knowledge (β = -1.743; P-value = 0.024). Additionally, there was a significant relationship between knowledge about vaccine and attitude to be immunization providers. Pharmacists with poor knowledge about vaccines had negative attitude to be immunization providers as compared to those with high knowledge (β = -2.631; P-value = 0.002). Furthermore, a significant number of the community pharmacists reported facing critical barriers to provide vaccines including legal liability, lack of personal resources and lack of appropriate training.

**Conclusion:**

Comprehensive training and certification programs for pharmacists are crucial to improve their competencies in handling and administering vaccines to increase the rate of vaccinations in Saudi Arabia.

## Introduction

Vaccines have helped in lowering deaths and complications related to infectious diseases, worldwide. According to the estimates by the World Health Organization (WHO), around 3.5 to 5 million deaths each year are prevented by vaccines [1]. Vaccines prevent deaths from more than twenty serious diseases including diphtheria, tetanus, influenza and measles [1]. Vaccines also help in control, prevention and reduction in mortality with infectious diseases such as COVID-19, H1N1 and influenza [2, 3] and are considered among the most effective public health interventions [3], However, dearth of information, worries about side effects, false beliefs and, hesitancy towards vaccinations have led to sub-optimal immunization rates in many countries around the world [4, 5]. WHO immunization agenda 2030 aims to increase vaccinations rates, and to decrease inequality in immunizations worldwide [2]. Community pharmacists had important role in increasing vaccinations rates especially in countries where they offer vaccination services and administration [6, 7].

In order to combat the issue of sub-optimal immunization, involvement of pharmacists as a non-traditional immunization provider was strategically adopted by several countries [8]. Various studies have explored the outcomes of vaccination services implementation at community pharmacies and reported that involvement of a pharmacist can be beneficial to increase the vaccine availability, accelerate the immunization and coverage rate and, effective patient education. All of these can eventually lead to prevention of new cases, and reduction in complications, thereby resulting in cost savings to the healthcare system [9].

A significant step in this regard was taken by the United States, the United Kingdom, Australia, Argentina, South Africa and the Philippines by providing legal authorization to community pharmacists to provide vaccination services [7, 8]. In the United States, pharmacists’ involvement in vaccination services has increased the rates of vaccinations [7]. During COVID-19, along with many countries, Saudi Arabia launched the initiative of providing vaccination service at community pharmacies to increase the vaccination rate in a short period of time [10, 11]. Although, numerous studies have recommended that the benefits of pharmacist involvement in vaccination provision outweighs the risk, it is imperative to evaluate whether pharmacists have a positive inclination and are willing to partake in provision of vaccination services which can provide a baseline impression before any change in policies [12–16].

In Poland, most of 1,777 pharmacists reported numerous benefits of availability of vaccination service at community pharmacies [12]. A Lebanese study found that most of the participants (n = 412) have good knowledge of vaccinations, and they are willing to provide vaccines in pharmacies [16]. In Saudi Arabia, a study that included 139 community pharmacies from Riyadh city reported that they are willing to deliver vaccination services at their pharmacies [15].

The inclusion of community pharmacists as a provider of vaccination service has yielded positive outcomes in many countries and other studies have supported the inclusion community pharmacists as immunization providers. Although, Saudi Arabia implemented this service during the COVID-19 pandemic and witnessed fruitful results, it is yet to upscale the service to include other vaccinations across the country. The existing body of literature currently lacks large scale studies that elicit the knowledge and attitude of community pharmacists toward vaccinations and identification of barriers that may hinder the provision of immunization service across the country. Hence, the results of the present study can be of significant value to the Saudi healthcare authorities and policy makers to gauge the readiness of community pharmacists and to devise corrective measures if needed, before any policy amendments and its implementation.

Therefore, we aimed to assess community pharmacists’ knowledge and attitudes toward vaccinations, their attitudes and readiness to be immunization providers and reported barriers for providing immunization service at their pharmacies in Saudi Arabia.

## Methods

### Study design

An online questionnaire-based, cross-sectional study was designed using convenience sampling (Snowball technique) to obtain community pharmacists’ responses across Saudi Arabia. In order to include participants across the geographical regions, twenty-two data collectors were assigned the responsibility for data collection. Five of the study authors coordinated with the data collectors throughout the data collection period. Initially, each data collector was asked to select five participants based on appropriate representation of gender, practice setting, job title, area and, experience. After the first set of five participants had completed the survey, they were requested to forward the survey link to five more participants who they deemed to fit the eligibility criteria and so on. The questionnaire was hosted online through Qualtrics XM and the responses were collected from 27^th^ May 2023 until 16^th^ August 2023. Licensed community pharmacists working at community pharmacies (chain/independent) in Saudi Arabia were eligible to participate in the study. The respondents who did not provide consent, were not registered pharmacists, and not working as community pharmacists were excluded.

### Study population

Licensed community pharmacists practicing in community pharmacies across varied geographical locations across Saudi Arabia.

### Sample size

For this purpose, we used an online sample size calculator (Raosoft®) [17]. The population of practicing community pharmacists in Saudi Arabia were estimated to be 8409 [18]. Sample size of 368 was obtained where the margin of error, confidence level and response distribution were set at 5%, 95% and 50% respectively. The estimated sample size was cross-verified by using another sample size calculator, Open Epi [19] which also revealed sample size as 368.

### Measures

A self-administered questionnaire which consisted of closed-ended questions was constructed after in-depth scrutiny of literature and was subsequently used to elicit responses from community pharmacists. A focus group comprising twenty community pharmacists were used as sample pilot to assess the content and face validity of the questionnaire. The questionnaire was in the English language and was composed of six sections. Questions pertaining to demographic information were included in the first section. Seven items in the second section assessed the attitude of community pharmacists towards vaccines. The third section assessed the attitude of community pharmacists to be immunization providers and consisted of six items. The fourth section evaluated the readiness of community pharmacists to be immunization providers with the help of five items. The fifth section comprised of sixteen items to test the knowledge of community pharmacists pertaining to vaccines and immunization. The sixth section had ten items which focused on identification of barriers for providing immunization service by community pharmacists at their pharmacies. The study questionnaire was prepared after systematic consideration and a wide-ranging review of related literature [12, 15, 16, 20, 21] by all authors. Items in sections three, four and five of the questionnaire were adopted from Edwards et al. [20], whereas those in section six were adopted from Capurso and Powers [21] (S1 Questionnaire).

### Data analysis

Data analyses were carried out using statistical software, STATA 18.0 (Stata, College Station: StataCorp LLC) and RStudio (RStudio Team, 2020). For each scale in the study, an exploratory factor through principal component extraction analysis was conducted. Barlett’s test was utilized to assess the appropriateness of data for factor analysis and to measure the sampling adequacy Kaiser-Meyer-Olkin test was applied [22]. Velicer’s test was conducted to determine the number of components [23]. Cronbach’s Alpha was used to evaluate the reliability of the instrument. If the result exceeds the threshold value of 0.7, the scale was considered reliable. S1 Table shows factor analysis and reliability tests for all the scales used in this study. All scales consisted of a single underlying factor. Descriptive statistics were used to express the sample characteristics and reported as frequencies, total percentages, means, and standard deviations. T-tests were computed to assess the relationships between continuous variables and outcomes. Multiple linear regression models were computed to assess the adjusted relationships between the explanatory variables (attitude toward vaccines and attitude to be immunization provider). The final model included sex, area of living, pharmacy type, experience, working hours, nationality, and knowledge about vaccines. The alpha level of p<0.05 was used to determine statistical significance.

### Ethical considerations

Before commencement of the study, the protocol along with the questionnaire was reviewed and approved (No: REC-44/10/650) by the Standing Committee for Scientific Research (HAPO-10-Z-001) at Jazan University, Saudi Arabia. Participants who received the questionnaire link were briefed with the study’s objective, main goals, benefits and risks of participating in the study in a separate section that pertained to informed consent. All participants were required to provide their consent by answering a specific question which asked them whether they have read and understood the study’s objective, goals, benefits and risks and click one of the suitable options (agree to participate in the study/disagree to participate in the study). All participants were required to provide their consent in the form before proceeding to the questionnaire section. The survey would end for those who did not agree to participate in the study. Withdrawal from the study was voluntary and study participants who provided their consent could also withdraw at any time. Confidentiality and anonymity of data was ensured for study participants.

## Results

### Sample characteristics

Our sample comprised of 384 community pharmacists (S1 Data). The mean age of the participants was 30.6 (SD = 4.5). Of the study sample (Table 1), 81% had bachelor’s in pharmacy or Doctor of Pharmacy degree while 18% had a postgraduate diploma. Likewise, 81% were males and 25% of respondents practiced in rural areas. Most of participants practiced pharmacy for 6 years or more. However, most of them were non-Saudis (59.6%).

**Table 1:** Study sample characteristics (n = 384).

Variable	n (%)
**Education Level**	
Bachelor/PharmD	259 (67.4)
> = Diploma	125 (32.6)
**Sex**	
Male	313 (81.5)
Female	71 (18.5)
**Area of Living**	
Urban	287 (74.7)
Rural	97 (25.3)
**Pharmacy Type**	
Independently Owned Pharmacy	36 (9.4)
Chain Pharmacy	348 (90.6)
**Job Title**	
Owner	9 (2.3)
Manager	39 (10.2)
Staff Pharmacist	299 (77.9)
Clinical Pharmacist	37 (9.6)
**Work Experience**	
Less than 1 year	39 (10.2)
1–5 years	162 (42.2)
6–10 years	117 (30.5)
>10 years	66 (17.2)
**Working hours per week**	
<10 hours	59 (15.4)
11–24 hours	20 (5.2)
25–40 hours	72 (18.8)
>40 hours	233 (60.7)
**Nationality**	
Saudi	155 (40.4)
Non-Saudi	229 (59.6)
**Knowledge about vaccines**	
Good	32 (8.4)
Average/moderate	146 (38.1)
Poor	205 (53.5)

### Knowledge about vaccines

The participants were asked sixteen questions (S1 Questionnaire) to assess their knowledge about vaccines. The response scale was true, false and don’t know. We categorized the participants into three categories based on the Blooms’ cut-off point [24]: good knowledge (≥80% correct answers); average/moderate knowledge (60–79% correct answers); and poor knowledge (<60% correct answers). Most of the participants had poor knowledge about vaccines (54%). Only 8.4% of the participants had good knowledge about vaccines.

### Attitude toward vaccines scale

**Fig 1:**
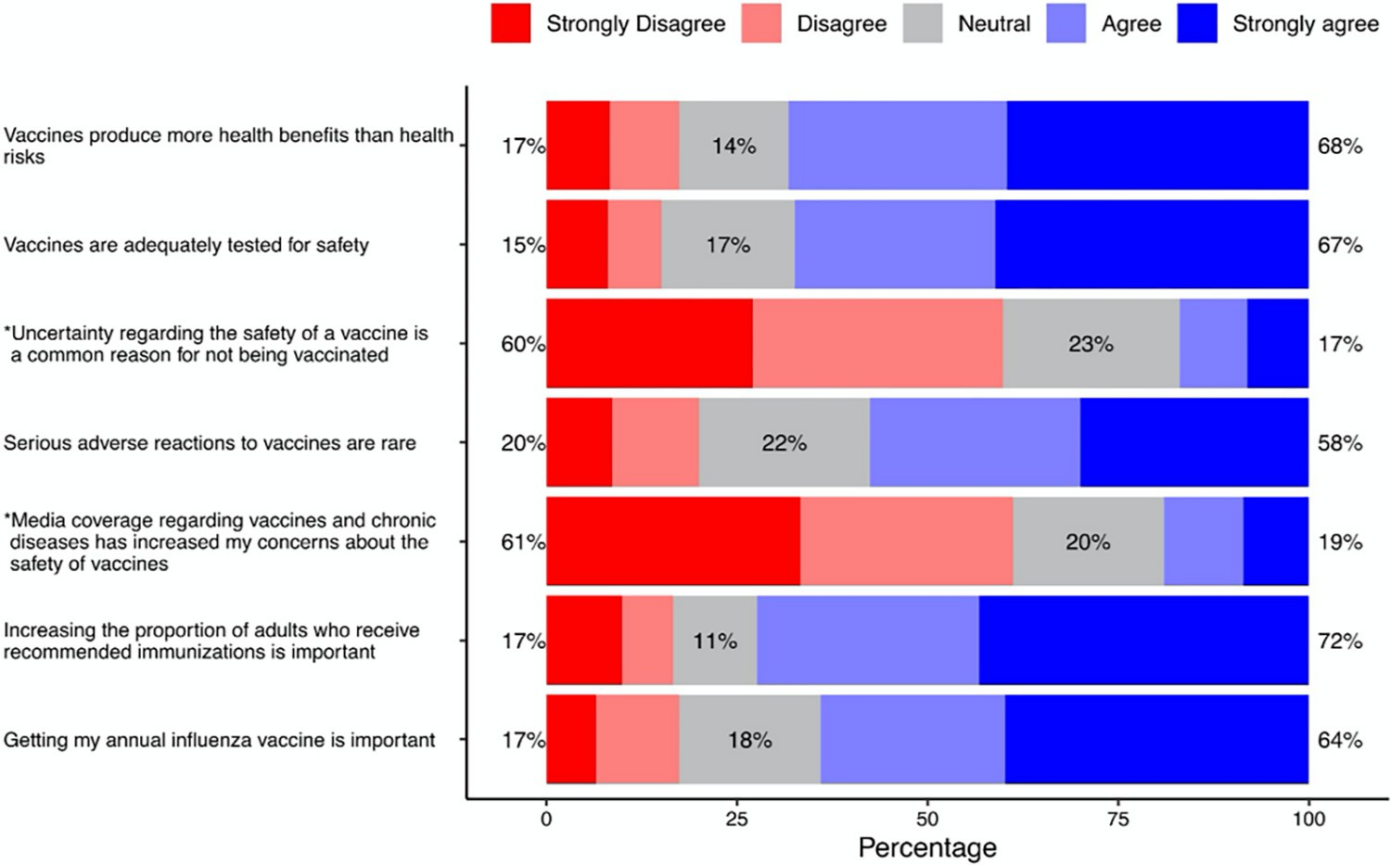
Attitude toward vaccines. *Negative statement: reverse coded.

A 7-item scale measured the attitude of community pharmacists toward vaccines. Likert-type scale was used to measure the responses ranging from “strongly disagree” to “strongly agree”. Only a single underlying factor was indicated by factor analysis. The Cronbach’s alpha for the scale was 0.91, suggesting that the items have high internal consistency. The mean score for attitude toward vaccines scale was 23.7 (SD = 4.1). Fifty-nine percent of the participants scored above the mean. Most of the participants (72%) either responded agree or strongly agree to the statement “Increasing the proportion of adults who receive recommended immunizations is important.” Likewise, most of them (68%) agreed or strongly agreed to the statement “Vaccines produce more health benefits than health risks.” Nevertheless, most of the pharmacists (61%) either disagreed or strongly disagreed to the statement “Media coverage regarding vaccines and chronic diseases has increased my concerns about the safety of vaccines.” Fig 1 shows the results regarding pharmacists’ attitude toward vaccine.

### Explanatory variables and attitude toward vaccines

Parameter estimates of the explanatory variables from an ordinary least squares regression on attitudes toward vaccines are illustrated in Table 2. There was a significant relationship between pharmacy location and attitude toward vaccines. Pharmacists who practiced in rural areas had negative attitude toward vaccines as compared to those who practiced in urban areas (β = -1.188; P-value = 0.017). Additionally, pharmacists with poor knowledge about vaccines had negative attitude toward vaccines as compared to those with high knowledge (β = -1.743; P-value = 0.024). On the other hand, pharmacists who worked 25 hours or more per week had significantly positive attitude than those who worked 10 hours or less.

**Table 2:** Parameter estimates of the explanatory variables from ordinary least squares regression on attitude towards vaccine among community pharmacists in Saudi Arabia (n = 384).

Explanatory Variable	β	95% Confidence Interval	P-Value
Sex			
Male	Reference		
Female	-0.248	(-1.567–1.071)	0.712
Area			
Urban	Reference		
Rural	-1.188	(-2.164 –-0.212)	0.017
Pharmacy Type			
Independently Owned Pharmacy	Reference		
Chain Pharmacy	0.023	(-1.536–1.581)	0.977
Experience			
Less than 1 year	Reference		
1–5 years	-0.48	(-1.862–0.902)	0.495
6–10 years	-1.053	(-2.697–0.591)	0.209
>10 years	-1.733	(-3.569–0.103)	0.064
Woeking Hours			
<10 hours	Reference		
11–24 hours	0.647	(-0.938–2.232)	0.423
25–40 hours	1.974	(0.3–3.647)	0.021
>40 hours	2.329	(0.884–3.774)	0.002
Nationality			
Saudi	Reference		
Non- Saudi	1.08	(-0.03–2.19)	0.056
Knowledge about vaccines			
High	Reference		
Moderate/Average	-0.943	(-2.432–0.547)	0.214
Poor	-1.743	(-3.255 –-0.231)	0.024

### Attitude to be immunization provider

**Fig 2:**
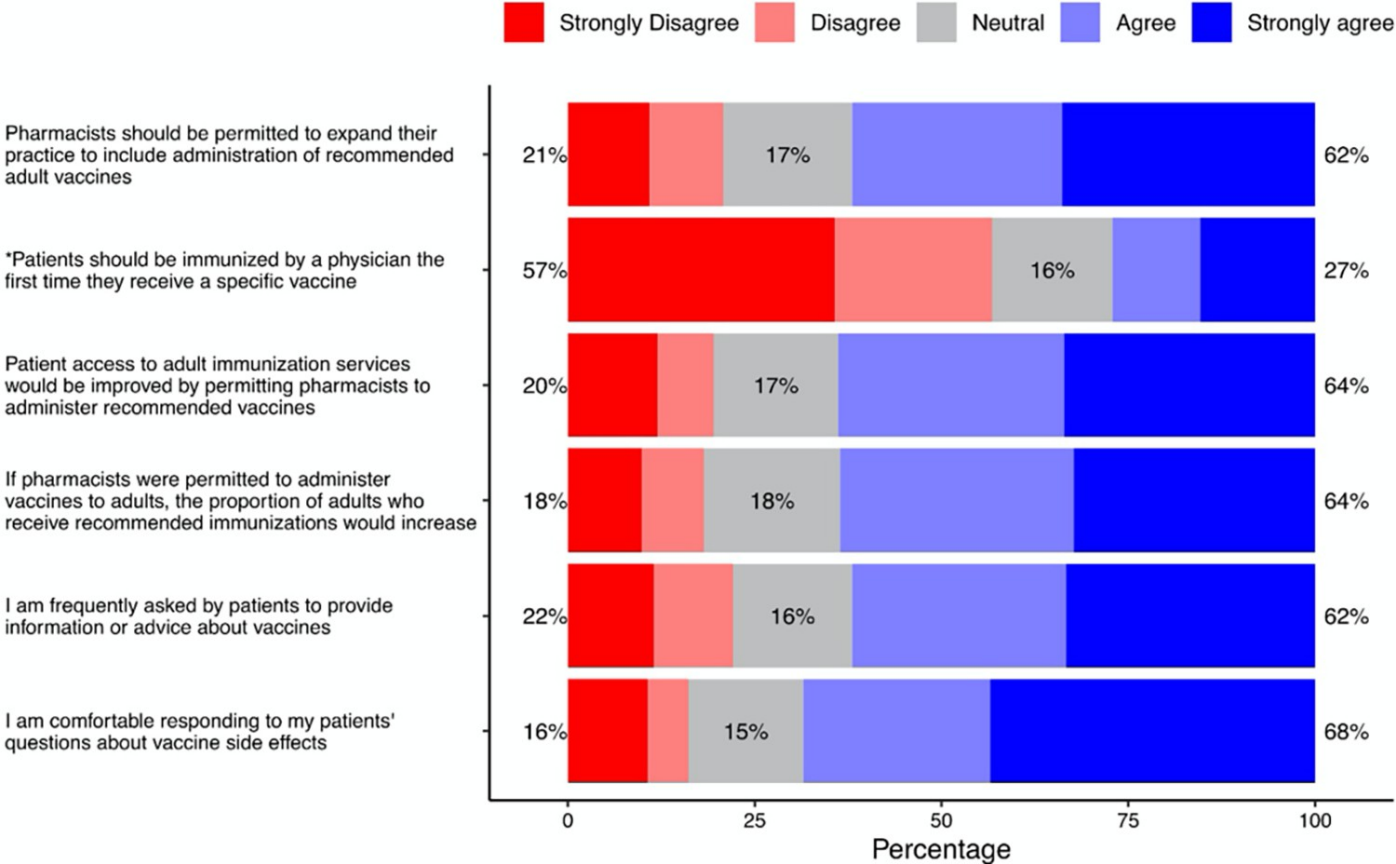
Attitude to be immunization providers. *Negative statement: reverse coded.

A 6-item scale was used to measure the community pharmacists’ attitude be immunization providers. The response scale was a Likert-type scale (“strongly disagree” to “strongly agree”). Factor analysis indicated a single underlying factor. High internal consistency was observed as the Cronbach’s alpha for the scale was 0.89. The mean score for attitude toward vaccines scale was 20.9 (SD = 5.0). Sixty-two percent of the participants scored above the mean. Most of the participants (68%) either responded agree or strongly agree to the statement “I am comfortable responding to my patients’ questions about vaccine side effects.” Likewise, most of them (64%) agreed or strongly agreed to the statement “If pharmacists were permitted to administer vaccines to adults, the proportion of adults who receive recommended immunizations would increase.” However, most of the pharmacists (57%) either disagreed or strongly disagreed to the statement “Patients should be immunized by a physician the first time they receive a specific vaccine.” Fig 2 shows the results regarding pharmacists’ attitude to be immunization providers.

### Explanatory variables and attitude to be immunization provider

Parameter estimates of the explanatory variables from an ordinary least squares regression on attitude to be immunization providers are shown in Table 3. There was a significant relationship between pharmacy location and pharmacists’ attitude to be immunization providers. Pharmacists who practiced in rural areas had negative attitude to be immunization providers as compared to those who practiced in urban areas (β = -1.335; P-value = 0.029). Additionally, pharmacists with poor knowledge about vaccines had negative attitude to be immunization providers as compared to those with high knowledge (β = -2.631; P-value = 0.002). On the other hand, pharmacists who worked 11 hours or more per week had a significantly positive attitude than those who worked 10 hours or less.

**Table 3:** Parameter estimates of the Explanatory variables from ordinary least squares regression on attitude being immunization provider among community pharmacists in Saudi Arabia (n = 384).

Explanatory Variable	β	95% Confidence Interval	P-Value
Sex			
Male	Reference		
Female	-0.271	(-1.887–1.345)	0.742
Area			
Urban	Reference		
Rural	-1.335	(-2.532 –-0.139)	0.029
Pharmacy Type			
Independently Owned Pharmacy	Reference		
Chain Pharmacy	-0.023	(-1.951–1.905)	0.982
Experience			
Less than 1 year	Reference		
1–5 years	-1.555	(-3.371–0.261)	0.093
6–10 years	-1.175	(-3.379–1.029)	0.295
>10 years	-2.028	(-4.437–0.381)	0.099
Woeking Hours			
<10 hours	Reference		
11–24 hours	3.849	(1.657–6.04)	0.001
25–40 hours	2.613	(0.726–4.501)	0.007
>40 hours	3.293	(1.641–4.945)	<0.001
Nationality			
Saudi	Reference		
Non- Saudi	0.558	(-0.93–2.045)	0.461
Knowledge about vaccines			
High	Reference		
Moderate/Average	-1.507	(-3.185–0.17)	0.078
Poor	-2.631	(-4.305 –-0.957)	0.002

### Pharmacists’ readiness to be immunization providers

**Fig 3:**
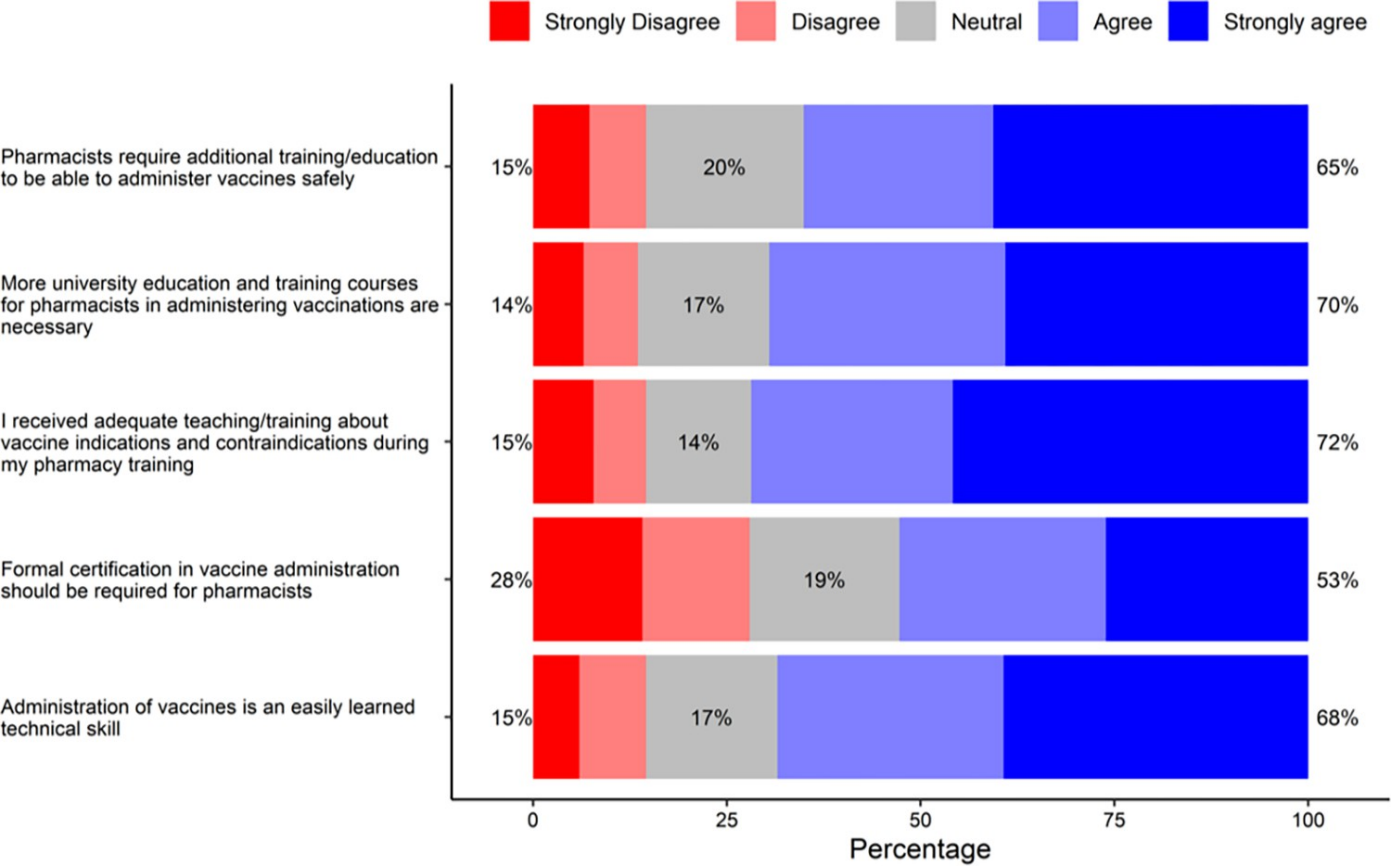
Readiness to be immunization provider.

Fig 3 illustrates the results regarding pharmacists’ readiness to be immunization providers. A 5-item scale was used to assess pharmacists’ readiness to be immunization providers. Likert-type scale (“strongly disagree” to “strongly agree”) was used to measure the responses and the factor analysis indicated a single underlying factor. The scale showed good internal consistency as the Cronbach’s alpha was 0.83. Most participants (72%) either agreed or strongly agreed to the statement “I received adequate teaching/training about vaccine indications and contraindications during my pharmacy training.” However, most of them (70%) agreed/strongly agreed with the statement “More university education and training courses for pharmacists in administering vaccinations are necessary.”. Also, majority in the sample (65%) agreed/strongly agreed with the statement “Pharmacists require additional training/education to be able to administer vaccines safely.”

### Barriers to provide immunization in community pharmacy

**Fig 4:**
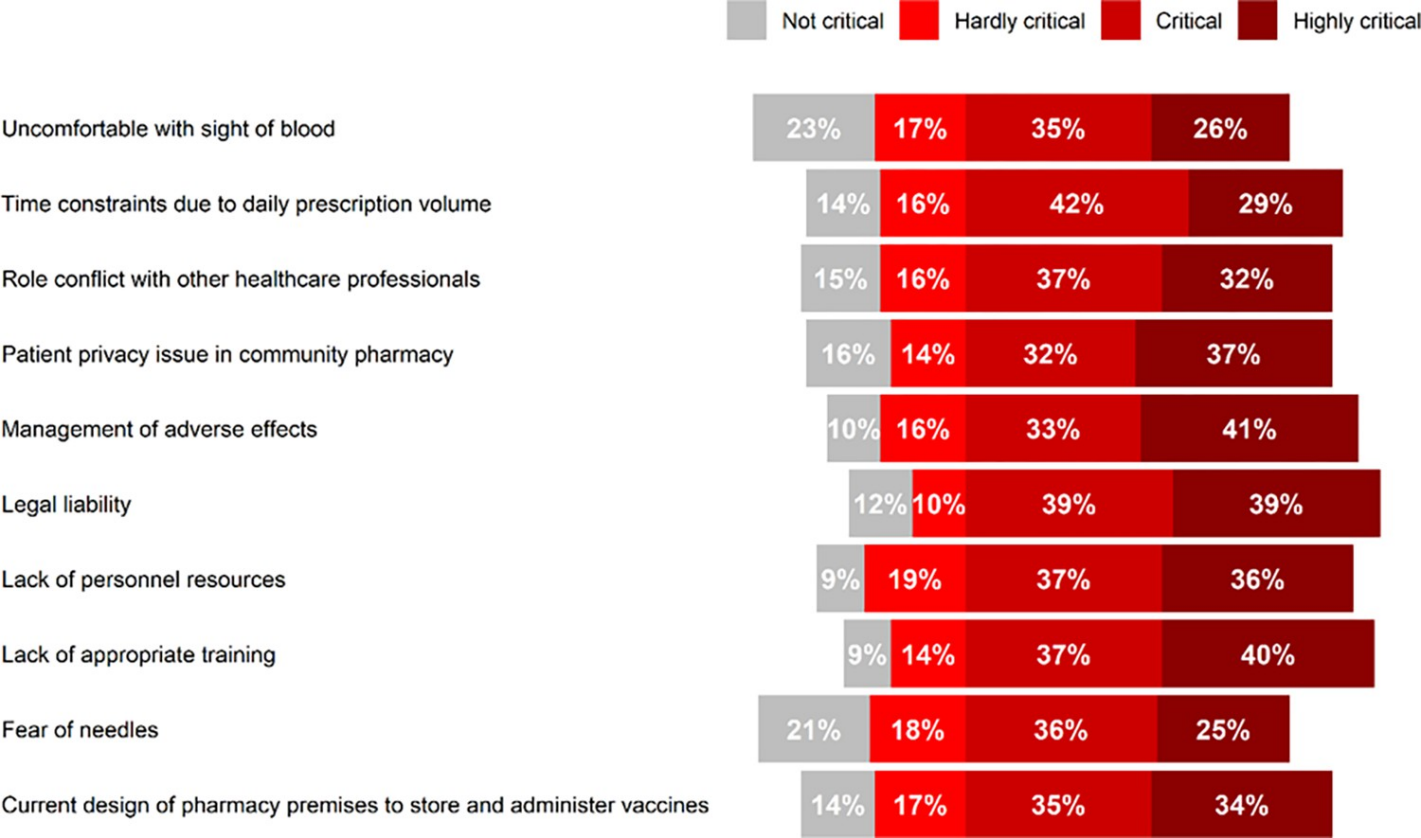
Barriers to provide immunization at community pharmacies.

Pharmacists were asked to rate ten statements on barriers for providing immunization at community pharmacies on a 1–4 Likert scale (not critical, hardly critical, critical, highly critical). Pharmacists reported “legal liability” as the most important barrier since most of them rated this barrier as critical (39%) or highly critical (39%). Additionally, the following barriers were rated as highly critical by more than 35% of the community pharmacists: “patient privacy issue in community pharmacy”, “management of adverse effects”, “lack of personal resources”, and “lack of appropriate training.” Fig 4 displays the barriers for providing immunization at community pharmacies.

## Discussion

The current study has examined knowledge and attitude and community pharmacists’ willingness to provide vaccines in Saudi Arabia. The role of community pharmacists is advancing in the Saudi’s healthcare system and they can contribute to infectious disease control programs by engaging in vaccination services which was evident during the COVID-19 pandemic. Community pharmacies in Saudi Arabia played a crucial role in providing the COVID-19 vaccination service [25, 26] and have demonstrated an effective model for implementing vaccination service for other infectious diseases. However, it has been shown that willingness to engage in these services is linked directly with the pharmacists’ knowledge of vaccinations [13].

Over half of the community pharmacists in our sample had poor knowledge and only a meagre (less than 10%) had good knowledge of vaccines. This is a concerning finding as other studies have reported varied levels of knowledge in their sample. Studies from Lebanon and Ethiopia reported contrasting results wherein more than 90% of the pharmacists in their sample had good knowledge of vaccines [16, 27]. Another study from Malaysia reported moderate knowledge level among half of the participating pharmacists [28]. Our findings related to the pharmacists’ knowledge of vaccination underscores a crucial gap which needs attention of healthcare authorities to implement programs that focus on improving the vaccine knowledge base of pharmacists.

The results indicated that community pharmacists in the study sample have positive attitude toward vaccines and are willing to provide vaccination services which was consistent with previous studies [12, 15]. However, we found negative attitudes among pharmacists who practiced in rural areas which may be attributed to lack of accessibility to continuous education programs and appropriate conditions. The responses of pharmacists on their role as immunization providers also reflected positive attitudes. Most pharmacists reported being comfortable at responding to patient queries and the immunization providers role can effectively lead to increased rate of vaccination among eligible individuals in the community. Evidence from a meta-analysis demonstrated that immunization rate substantially increased with pharmacists as immunization providers. Moreover, developed countries such as United States, United Kingdom and Canada have worked towards granting autonomy to pharmacists in administering vaccines as this has proved to improve health outcomes and reduction in healthcare costs [28]. We also report significant association between knowledge and pharmacists’ attitude to be immunization providers where poor knowledge was associated with negative attitude. Similar findings were reported from studies conducted in United States and New Zealand among healthcare professionals [29, 30].

Responses related to the readiness of pharmacists to be immunization providers showed that around three-quarters of pharmacists reported receiving adequate training about vaccines in their pharmacy program. However, around half of participating pharmacists reported receiving sufficient training in Lebanon and Malaysia [16, 28]. Additionally, most of them reported the necessity of additional educational and training courses at the university level in regard to vaccine administration, and that pharmacists require further training and/or education to be proficient in safe vaccine administration. Comprehensive training and certification programs for pharmacists are crucial to improve their competent in handling and administering vaccines [31]. Moreover, such programs can help in increasing the rates of vaccinations in rural and underserved communities.

Nevertheless, a significant number of the community pharmacists reported facing critical barriers to provide vaccines including legal liability, lack of personal resources and lack of appropriate training. As in our study, many other have reported legal liability as the most critical barrier in provision of immunization service at community pharmacies [20, 32–34]. However, other studies from Ethiopia and Jordan have reported lack of authorization as the most common barrier for providing vaccination service at community pharmacies [27, 35]. Therefore, it important to review and update the regulations in Saudi Arabia in regard to authorizing pharmacists to administer vaccines. Additionally, the government and health agencies need to establish systems for pharmacists to report vaccine administration and to enact regulations to provide legal protections for pharmacists administering vaccines. In addition, it is crucial to also identify the patient-related barriers that could impede implementation of vaccination-related services at community pharmacies [31].

The present study highlights the existing gap in pharmacists’ knowledge towards vaccines and necessitate urgent addressal by the Saudi healthcare authorities by implementing tailored educational programs for the community pharmacists. Moreover, our findings report that attitude be an immunization provider is significantly associated with the knowledge of pharmacists regarding vaccines, which underscores the crucial need of vaccine-based educational programs for community pharmacists in Saudi Arabia. Also, our study findings emphasize on the serious concern pertaining to negative attitudes of community pharmacists practicing in rural areas which can be of great hindrance to implement vaccination service across the country. Healthcare authorities need to place special focus on identifying the problems faced by community pharmacists in rural areas before designing educational programs. The results of the present study warrant attention of program leaders at pharmacy schools across Saudi Arabia to incorporate vaccine-related topics into the curriculum along with inclusion of vaccine administration in the experiential training to prepare future pharmacists that are knowledgeable in vaccines as well as adept in vaccine administration. Overall, the results of the present study can aid the healthcare authorities and policy makers by providing important insights regarding the existing knowledge, attitudes and readiness of community pharmacists and the reported barriers for implementing vaccination service, and addressal of prevailing issues is paramount to advance the scope of the community pharmacist to be an immunization provider in Saudi Arabia. Nevertheless, further research is necessitated to investigate the impact of educational and policy interventions for improving knowledge and addressing the identified barriers among community pharmacists in Saudi Arabia

The current study has limitations. As we employed a self-administered questionnaire, possibility of social desirability bias exists. Also, inference of causality is not possible due to the cross-sectional nature of the study. Use of structured interviews would be more appropriate to elicit the responses from the participants. Also, due to the non-random nature of snowball sampling, the study methodology can be liable to sample bias as the respondents were chosen through referrals. In addition, limited generalizability is a limitation of snowball sampling due to which the study findings may not be representative of the larger population. Community bias is also another limitation of snowball sampling as the initial set of participants would have a greater impact on the sample.

## Conclusion

The present study findings shed light on the current scenario regarding the community pharmacists’ knowledge, attitudes and willingness to provide vaccination services in Saudi Arabia. Our study revealed poor knowledge of community pharmacists about vaccines and underlines the necessity of continuing education programs and trainings for community pharmacists to improve their knowledge base of vaccines. Nonetheless, our findings demonstrate community pharmacists’ positive attitudes towards vaccines, their role as an immunization provider and readiness to be immunization providers. These encouraging findings demonstrate the willingness of community pharmacists to expand their scope of practice which highlight the potential for implementation of vaccine services at community pharmacies. Also, pharmacist-related barriers identified in our study provide valuable insights to the health authorities and policy makers for designing measures to support the vaccination efforts of community pharmacists. By improving knowledge and expertise, community pharmacists can be confident and equipped to make significant contribution in achieving national vaccination goals. Examples from real-world scenarios have shown the advantages of involving community pharmacists in vaccination efforts, with significant implications for clinical practice. By granting them the authority to administer vaccines, healthcare authorities can leverage the expertise of community pharmacists to boost vaccination rates, ultimately contributing to improved public health outcomes. As the most accessible healthcare professionals, community pharmacists can provide greater convenience and more opportunities for vaccination, leading to increased coverage and a decrease in the occurrence of vaccine-preventable diseases. From the perspective of healthcare authorities, extending the role of community pharmacists in immunization can reduce the strain on public health centers and hospitals, enable more effective utilization of healthcare resources, and result in cost savings by preventing disease outbreaks and reducing hospitalizations.

## Supporting information

S1 Data (XLSX)

S1 Questionnaire (PDF)

S1 Table (PDF)
