# Supplementary material for: Assessment of community pharmacists’ knowledge, attitudes and their willingness to provide vaccination services in Saudi Arabia
Source: PLoS One. 2024 May 28;19(5):e0304287. doi: 10.1371/journal.pone.0304287 (PMC11132504; doi:10.1371/journal.pone.0304287)
Supplement: S1 Questionnaire — (PDF) [file pone.0304287.s002.pdf]

Assessment of community pharmacists' knowledge, attitudes and their willingness to provide vaccination services in Saudi Arabia

Greetings! You are invited to participate in a study being conducted by a research team from College of Pharmacy, Jazan University and King Fahd Central Hospital, Jazan. The purpose of the study is to **assess the community pharmacists' knowledge, attitudes and their willingness to provide vaccination services in Saudi Arabia.**

Please read the information below related to your rights pertaining your participation in this research study

PARTICIPATION
Your participation in this survey is voluntary. You may refuse to take part in the research or you may skip any question you do not wish to answer for any reason.

BENEFITS & RISKS
You will receive no direct benefits from participating in this research study. However, your responses may help us learn more about the knowledge, attitudes and willingness of community pharmacists to provide vaccination services. There are no foreseeable risks involved in participating in this study other than those encountered in day-to-day life.

CONFIDENTIALITY
Your survey answers will be stored initially with Google Forms in a password protected electronic format. Data will later be downloaded and stored on secure servers of College of Pharmacy, Jazan University.

No names or identifying information would be included in any publications or presentations based on these data, and your responses to this survey will remain confidential.

CONTACT
If you have questions concerning the study, contact the principal investigator by email at ameraya@jazanu.edu.sa

**Are you a licensed community pharmacist working at a chain or independent community pharmacy in Saudi Arabia**	☐ **YES**<br>☐ **NO**
Please indicate by checking the box below that that you have read and agree to the above information before proceeding to the questionnaire<br>.<br>**I have read and understand the previous information and agree to participate in this research study.**	☐ **YES**<br>☐ **NO**

Assessment of community pharmacists' knowledge, attitudes and their willingness to provide vaccination services in Saudi Arabia

QUESTIONNAIRE

Demographics
1) **Age** ……… years
2) **Gender** ☐ Male ☐ Female
3) **Education** ☐ Bachelors ☐ PharmD ☐ Masters ☐ Ph.D
4) **Please indicate your region (province) of residence.**<br>☐ Makkah ☐ Madinah ☐ Riyadh ☐ Eastern Province ☐ Asir ☐ Baha ☐ Jazan ☐ Jawf ☐ Tabuk ☐ Ha'il ☐ Northern Borders ☐ Qasim ☐ Najran
5) **Please indicate your area of residence** ☐ Urban ☐ Rural
6) **Where do you primarily practice pharmacy?** ☐ Independently owned pharmacy ☐ Pharmacy chain
**7) Please indicate your job status** ☐ Owner ☐ Manager ☐ Staff Pharmacist ☐ Clinical Pharmacist
**8) Please indicate your experience (in years) as a pharmacist** ☐ < 1 year ☐ 1 to 5 years ☐ 6 to 10 years ☐ >10 years
**9) Please indicate your weekly working hours** ☐ Less than 10 hours ☐ 11 to 24 hours ☐ 25 to 40 hours ☐ > 40 hours
**10) Please indicate your nationality** ☐ Saudi ☐ Non-Saudi

**Attitude Towards Immunization**					
**Please indicate your agreement to the following statements.**	**Strongly Disagree**	**Disagree**	**Neutral**	**Agree**	**Strongly Agree**
1. Increasing the proportion of adults who receive recommended immunizations is important.	①	②	③	④	⑤
2. Media coverage regarding vaccines and chronic diseases has increased my concerns about the safety of vaccines.	①	②	③	④	⑤
3. If I do not receive the influenza vaccine each year, I am at risk of contracting influenza and then can spread it to my patients.	①	②	③	④	⑤
4. Uncertainty regarding the safety of a vaccine is a common reason for not being vaccinated.	①	②	③	④	⑤
5. Getting my annual influenza vaccine is important.	①	②	③	④	⑤
6. Vaccines produce more health benefits than health risks.	①	②	③	④	⑤
7. Serious adverse reactions to vaccines are rare.	①	②	③	④	⑤

**Attitude To be Immunizers**					
**Please indicate your agreement to the following statements.**	**Strongly Disagree**	**Disagree**	**Neutral**	**Agree**	**Strongly Agree**
1. Patients should be immunized by a physician the first time they receive a specific vaccine.	①	②	③	④	⑤
2. I am comfortable responding to my patients' questions about vaccine side effects.	①	②	③	④	⑤
3. I am frequently asked by patients to provide information or advice about vaccines.	①	②	③	④	⑤
4. If pharmacists were permitted to administer vaccines to adults, the proportion of adults who receive recommended immunizations would increase.	①	②	③	④	⑤
5. Patient access to adult immunization services would be improved by permitting pharmacists to administer recommended vaccines.	①	②	③	④	⑤
6. I received adequate teaching/training about vaccine indications and contraindications during my pharmacy training.	①	②	③	④	⑤
7. Pharmacists should be permitted to expand their practice to include administration of recommended adult vaccines.	①	②	③	④	⑤

Readiness of pharmacists to be immunizers					
**Please indicate your agreement to the following statements.**	**Strongly Disagree**	**Disagree**	**Neutral**	**Agree**	**Strongly Agree**
1. I received adequate teaching/training about vaccine indications and contraindications during my pharmacy training.	①	②	③	④	⑤
2. More university education and training courses for pharmacists in administering vaccinations are necessary.	①	②	③	④	⑤
3. Administration of vaccines is an easily learned technical skill.	①	②	③	④	⑤
4. Pharmacists require additional training/education to be able to administer vaccines safely.	①	②	③	④	⑤
5. Formal certification in vaccine administration should be required for pharmacists.	①	②	③	④	⑤

Knowledge about vaccines			
**Please indicate your agreement to the following statements.**	**True**	**False**	**I don't know**
1. Mild illness, with fever, is a reason to withhold vaccinations.	①	②	③
2. If a mother is breastfeeding, she should not be vaccinated.	①	②	③
3. Pregnant women who are expected to deliver during influenza season should receive influenza vaccine.	①	②	③
4. Anaphylactic reaction to a previous dose of vaccine is a contraindication to further doses of the same vaccine.	①	②	③
5. Unvaccinated people with mild symptoms of influenza can spread the disease to others.	①	②	③
6. Current scientific evidence supports an association between vaccines and multiple sclerosis.	①	②	③
7. Persons receiving immunosuppressive medications should not receive influenza vaccine.	①	②	③
8. Improper storage of vaccines may affect the immune response of the vaccine recipient.	①	②	③
9. Influenza vaccine should not be given during the first trimester of pregnancy.	①	②	③
10. Routine immunization should be delayed in individuals with moderate to severe illness, with or without fever.	①	②	③
11. Annual influenza immunization is recommended for all health care professionals in contact with individuals in high risk groups	①	②	③
12. The risk of an allergic reaction can be decreased by effective screening prior to vaccination.	①	②	③
13. Local adverse reaction such as pain, swelling, and redness at the injection site generally occurred within a few hours of the injection and are usually mild and self-limited.	①	②	③
14. Systemic adverse reactions may occur following receipt of live, attenuated vaccines which must replicate to produce immunity.	①	②	③
15. A systemic reaction is usually mild and occurs 3–21 days after the vaccine was administrated (incubation period of the vaccine)	①	②	③
16. Providers should report any clinically significant adverse event occurring after administration of the vaccine even if they are unsure whether the vaccine caused the event	①	②	③

THANK YOU FOR YOUR PARTICIPATION
