# Supplementary material for: Assessment of community pharmacists’ knowledge, attitudes and their willingness to provide vaccination services in Saudi Arabia
Source: PLoS One. 2024 May 28;19(5):e0304287. doi: 10.1371/journal.pone.0304287 (PMC11132504; doi:10.1371/journal.pone.0304287)
Supplement: S1 Table — (PDF) [file pone.0304287.s003.pdf]

Scale	Item	Single Factor Loadings	Cronbach's Alpha
**Attitude toward vaccines**	Vaccines produce more health benefits than health risks.	0.8271	0.91
	Vaccines are adequately tested for safety.	0.8544	
	Uncertainty regarding the safety of a vaccine is a common reason for not being vaccinated.	0.6654	
	Serious adverse reactions to vaccines are rare.	0.7046	
	Media coverage regarding vaccines and chronic diseases has increased my concerns about the safety of vaccines.	0.6814	
	Increasing the proportion of adults who receive recommended immunizations is important.	0.8207	
	Getting my annual influenza vaccine is important.	0.7785	
**Attitude to be immunization provider**	Patients should be immunized by a physician the first time they receive a specific vaccine.	0.5467	0.89
	I am comfortable responding to my patients' questions about vaccine side effects.	0.8230	
	I am frequently asked by patients to provide information or advice about vaccines.	0.7309	
	If pharmacists were permitted to administer vaccines to adults, the proportion of adults who receive recommended	0.8359	
	Patient access to adult immunization services would be improved by permitting pharmacists to administer	0.8281	
	Pharmacists should be permitted to expand their practice to include administration of recommended adult vaccines.	0.8018	
